# Supplementary material for: Emergence of superconductivity in the cuprates via a universal percolation process
Source: Nat Commun. 2018 Oct 18;9:4327. doi: 10.1038/s41467-018-06707-y (PMC6193991; doi:10.1038/s41467-018-06707-y)
Supplement: Supplementary file 1 — Supplementary Information [file 41467_2018_6707_MOESM1_ESM.pdf]

Supplementary Materials

Emergence of superconductivity in the cuprates via a universal percolation process

Pelc et al.

Supplementary Notes

Supplementary Note 1: Previous microwave studies and comparison of nonlinear response to vortex theory

An essential feature of our experiment is that it is performed without contacts on the sample, thereby eliminating possible nonlinearities in contact regions. Moreover, the contact-free measurement enables control of the sample temperature in a wide range without changing the temperatures of the excitation and detection coils. A special sample probe for use in superconducting magnets was constructed for this purpose. We note that some prior nonlinear microwave experiments[1-5], performed on YBCO, were interpreted either phenomenologically, or their importance for studying the emergence regime was not discussed in detail. Results of a phase-sensitive experiment at GHz frequencies[5] were modeled in terms of a field-based model dominated by the nonlinearity of the imaginary conductivity. Importantly, our MHz-range experiment is much closer to the zero-frequency limit than these prior studies, which minimizes the need for possible corrections from frequency-dependent and magnetoelectric effects.

Vortex dynamics has been extensively studied in type-II superconductors due to its technical importance. Linear and nonlinear harmonic responses have been calculated using phenomenological vortex electrodynamics appropriate for cuprate superconductors[6,7]. However, our results are incompatible with the calculations, for several reasons. According to those calculations, amplitudes of higher harmonics should diminish rapidly – third harmonic response should be significantly smaller than second harmonic (in a small constant external field of about H_{c1} ~ 10 G). To test this, we measured the second harmonic response, applying both an oscillating and a constant magnetic field (in the range from about 1 to 100 G). We only find a detectable second-harmonic peak in the cleanest sample, Hg1201, its width an order of magnitude smaller than the third harmonic, and the signal already below the detection limit in an external field of about 100 Gauss. This weak second-harmonic peak (orders of magnitude smaller than the third-harmonic peak) just above T_c is only detected in Hg1201, probably because of the fact that this cuprate exhibits relatively weak vortex pinning[8]; LSCO and YBCO-Zn feature prominent point-like disorder that can pin vortices and diminish their contribution to the nonlinear response. Moreover, all harmonics are predicted to diminish equally quickly with applied external field, and to disappear above the superconducting T_c. This prediction indeed

describes a separate reference measurement that we performed on a niobium single crystal (Supplementary Figure 1), but it is inconsistent with the observed universal third-harmonic behaviour in the cuprates. Although it is possible that a comparatively small vortex contribution is present very close to T_c (especially in Hg1201), where our minimal percolation model is invalid, the significant signal observed to relatively high temperatures cannot be due to vortices.

Supplementary Note 2: Relevance of 2D fluctuations

We note that the quasi-2D case of the Ginzburg-Landau conductivity (see Methods) – corresponding to Kosterlitz-Thouless physics – is readily obtained from first principles or by setting $\Lambda_{ab} >> \Lambda_c$. In that case, the agreement with measurements is even worse than in the anisotropic 3D case, which indicates that the prominent transport nonlinearities observed in $La_{1.875}Ba_{0.125}CuO_4$ (LBCO-1/8) and attributed to quasi-2D Kosterlitz-Thouless physics[9] are exceptional – nonlinear response cannot be said to universally originate from 2D fluctuations in the cuprates. A similar conclusion can be reached upon reexamining recent Seebeck coefficient data[10] for underdoped $La_{2-x-y}Eu_ySr_xCuO_4$ (Eu-LSCO). Although near T_c the emergence of superconductivity seems to be well described by a 2D Gaussian model[10], when the very same data are plotted on a linear temperature scale (Figure 4c in the main text) a clear exponential tail is observed that extends to higher temperatures. Importantly, in Eu-LSCO the superconducting transition is strongly suppressed and broadened[10] compared to LSCO, with T_c on the order of 5 K, rendering Eu-LSCO a rather unrepresentative cuprate. Its low T_c provides for a large accessible fluctuation range in relative temperature $(T - T_c)/T_c$, but on the absolute temperature scale the fluctuations extend only about $\Xi_0/2 \sim 15$ K above T_c, as in all other cuprates examined in our work. The experiments on Eu-LSCO are thus compatible with our conclusion that conventional GL fluctuations (with T_c as the relevant scale) are only visible very close to T_c, whereas further away from T_c inhomogeneity-induced superconducting percolation (with the universal Ξ_0 as the relevant scale) dominates. This is also clear from Figure 1c in the main text: close to T_c the percolation prediction deviates from the data, as expected when the fraction of superconducting patches is large. Yet in that regime the 2D theory as applied to Eu-LSCO may not be universally valid in the cuprates: Eu-LSCO has many similarities to LBCO-1/8, where charge stripes lead to dynamical layer decoupling and effective two-dimensionality[9,10]. Similar effects could plausibly occur in Eu-LSCO, and while certainly interesting in their own right, they cannot be said to be representative of all cuprates.

Supplementary Note 3: Percolation processes in other aspects of cuprate physics

The vast majority of theoretical models applied to the cuprates are based on the assumption of lattice translational invariance. Nevertheless, the effects of various types of disorder have been extensively discussed, including superconducting percolation and pseudogap inhomogeneity[11-24]. Experimental observations indicate that inherent inhomogeneity is prevalent in the cuprates, in related lamellar systems, and in perovskites in general[11-13]. For example, it is known from STM and nuclear quadrupolar resonance measurements[14-16,23,25] that significant nanoscale inhomogeneity exists already well above the pseudogap temperature. In fact, it has been argued that transport data are consistent with a temperature-dependent carrier localization that is inhomogeneous in real space[26,27]. In this picture, the pseudogap temperature T^* signifies a percolation transition involving CuO_2 units with one localized hole, and the localization process is complete at the slightly lower temperature T^{**}. This is also consistent with an extended analysis of STM data, relating high-energy inhomogeneous gaps to effective pseudogaps and superconductivity[24]. Doping and temperature have similar effects in this picture: the CuO_2 sheets evolve from x carriers deep in the pseudogap state to $1 + x$ carriers both at high hole-dopant concentrations and at high temperature. As a function of temperature, the inhomogeneous (de)localization must span a broad range of at least 1000 K in order to be consistent with both Hall effect and STM results[23,25,26]. In stark contrast, the superconducting heterogeneity scale Ξ_0 identified in the present work is only about 30 K. Within this picture, the localization of one carrier per CuO_2 unit renormalizes the underlying nanoscale inhomogeneity, giving rise to the smaller superconducting inhomogeneity scale Ξ_0 (which corresponds to a gap distribution with a width of about 3 meV).

Supplementary Note 4: Nonlinear response in a magnetic field

The field-dependent nonlinear response can be calculated in the percolation model if one assumes a dependence of J_c on external magnetic field. If the simplest, linear dependence is taken, the nonlinear response decreases exponentially with field. Empirically, the form of the σ_{3n} (H/H_0) master curve in Figure 3b is consistent with

$$\sigma_{3n}\left(H/H_0\right) = e^{-\left(H/H_0\right)^{1/2}} \quad (1)$$

where H_0 is the suppression field scale. We note that fitting the curves in Figure 3b in the main text with the more general functional form $\sigma_{3n}(H) = \exp\left[-(H/H_0)^\beta\right]$ yields an exponent somewhat larger than 1/2, β = 0.59 ± 0.07, and values of H_0 shifted upward by about 10% compared to the curve with β = ½ (see Supplementary Figure 4 for a comparison). Therefore, in order to obtain better agreement with experiment, rather than a linear dependence of the critical current on external field, the phenomenological form $J_c \sim const. - (H/H_0)^\beta$ should be used. However, such a simple treatment ignores the important fact that, due to phase coherence effects, a large superconducting cluster will have a different dependence of J_c on H than a small one. Thus a large field will render most clusters normal, except for the largest ones (including the sample-spanning cluster when $P > P_\pi$). This cannot be easily incorporated in the effective medium calculation and would require more elaborate lattice simulations, which we leave for future work. Importantly, due to the percolation physics, cluster-size effects are much more important when considering the magnetic field response than the zero-field gap inhomogeneity close to T_c. The cluster sizes vary over a wide range, which implies that cluster size and underlying (average) gaps are only weakly correlated. Importantly, the free energy of a cluster also depends on its size. Therefore, large clusters with somewhat smaller average gaps win over small clusters with larger gaps: the relative difference in gaps is roughly Ξ_0/T_c, which is, e.g., about 0.3 for optimally doped Hg1201, whereas the cluster sizes can differ by many orders of magnitude.

The experimental determination of nonlinear conductivity peak heights on external field is performed after the subtraction of the field-independent step-like "background" contribution (Supplementary Figure 5). For most samples, this step is small compared to the peak, and therefore the correction is minor. However, this is not the case for YBCO-Zn, probably due to the fact that it is a powder sample – in the percolation picture, the signal below T_c in a single crystal is due to the single sample-spanning cluster, whereas in a powder every grain contributes with its own grain-spanning cluster. Thus the relative intensity of the sample/grain-spanning clusters below T_c is significantly larger in a powder. The subtraction is performed by fitting a simple three-parameter sigmoidal form, $\sigma_{3n,step} = a/\left[1+\exp\left((T-T_{step})/b\right)\right]$, to the high-field data (Supplementary Figure 5) and then subtracting this contribution from the lower-field data. T_{step} is taken to have the same field dependence as T_c. We note that the subtraction is not performed in

the analysis of the zero-field temperature-dependent data (Figure 1 in the main text), since the correction is small on a logarithmic scale and only important close to the peak.

A quantitative comparison of our H_0 to H_{c2} values from the literature is more problematic than might seem, due to the large H_{c2} values typically encountered in the cuprates, and because the complicated temperature-field phase diagram renders an unambiguous identification of H_{c2} difficult. In the inset of Figure 3b in the main text, we compare our results for H_0 with H_{c2} determined from resistivity/heat conductivity measurements[28] and muon spin rotation[29] for YBCO. Without entering into the details of the H_{c2} determination and the assumptions involved there, we conclude that a qualitative agreement between the doping dependencies of H_0 and H_{c2} does exist. A more detailed quantitative comparison does not make sense anyway, since our effective medium calculation should only be viewed as a first approximation.

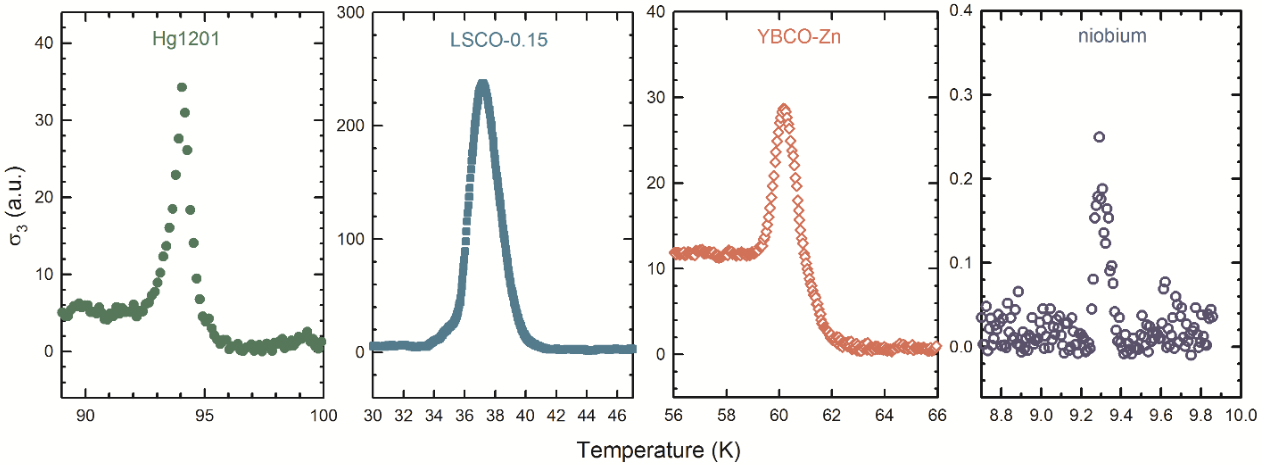

Supplementary Figure 1 | Raw third-harmonic data. Results for three cuprate samples are compared to a niobium single crystal. Even though a peak is visible for Nb as well, it is substantially narrower and weaker in intensity than for the cuprates – the peak signals for cuprate crystals of similar size are 2-3 orders of magnitude larger. The units are the same for all samples – note the difference in scale for the niobium measurement.

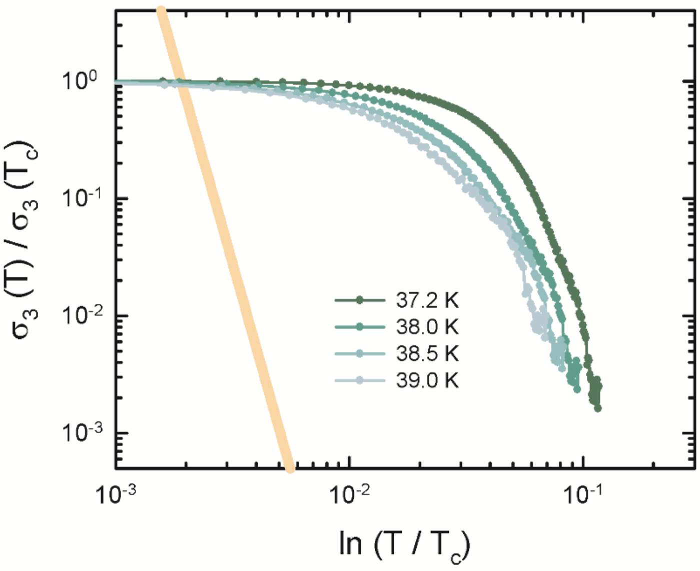

Supplementary Figure 2 | Effect of T_c shift on the nonlinear conductivity temperature dependence. In-plane nonlinear response of LSCO-0.15, plotted on the Ginzburg-Landau reduced temperature scale, for different choices of T_c. The value that corresponds to the peak in σ_{3n} is 37.2 K. The yellow line is the theoretical GL prediction, multiplied by a constant factor of 10 in an attempt to improve the agreement.

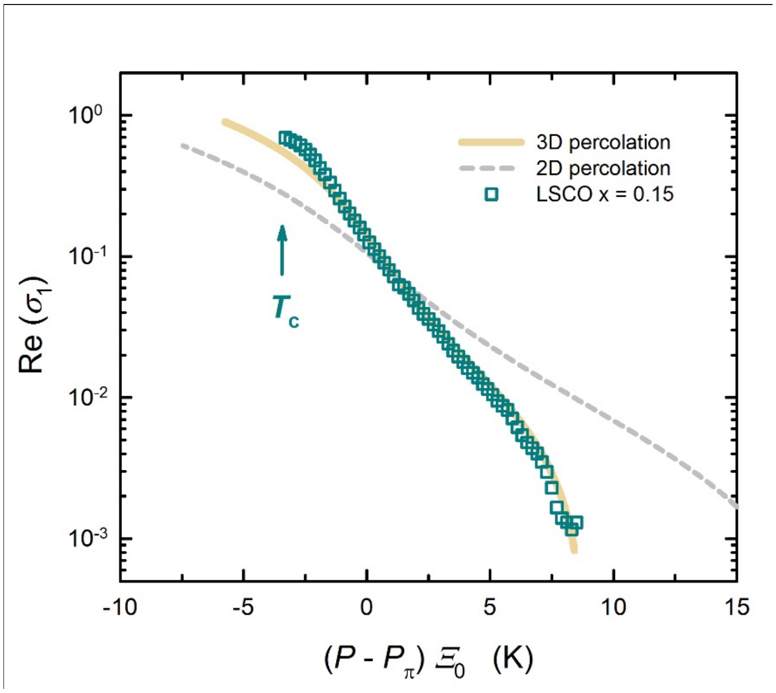

Supplementary Figure 3 | Modeling of linear conductivity. Comparison between data for LSCO (x = 0.15) and the results of 2D (dashed line, with P_π = 0.6) and 3D (full line, with P_π = 0.3) site percolation calculations. The same value Ξ_0 = 28 K (obtained from nonlinear conductivity) was used in both calculations. This comparison demonstrated that the data are compatible with 3D percolation.

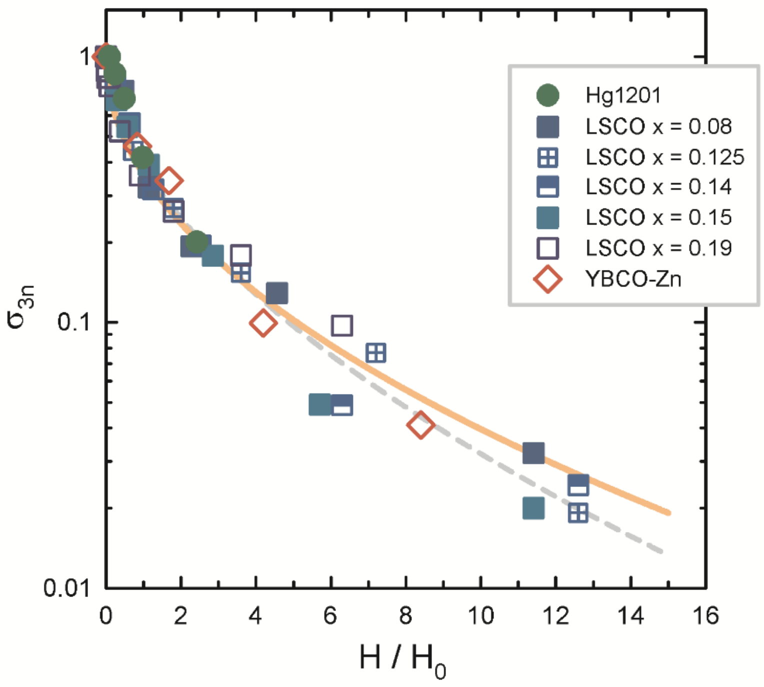

Supplementary Figure 4 | Magnetic field dependence of the nonlinear conductivity peak. Data are the same as in Figure 3 in the main text, with two different fit results. The solid line is the stretched exponential function with exponent ½, and the dashed line a best fit stretched exponential with exponent 0.59.

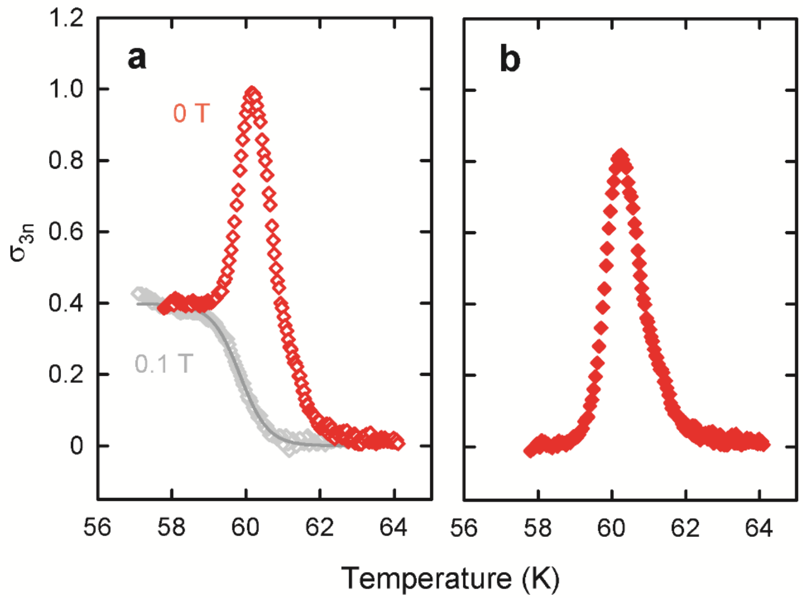

Supplementary Figure 5 | Step-like response subtraction for YBCO-Zn. a, Raw data in zero field and 0.1 T (where the peak is completely suppressed), with the fitted sigmoidal function (grey line). **b,** Zero-field data with 0.1 T data subtracted.

Supplementary References

[1] Leviev, G. I., Papikyan, R. S. & Trunin, R., Nonlinear microwave response of YBaCuO in a critical state. *J. Exp. Theor. Phys.* **99,** 359-362 (1990).

[2] Bolginov, V. V., Genkin, V. M., Leviev, G. I. & Ovchinnikova, L. V., Nonlinear microwave
[2] Bolginov, V. V., Genkin, V. M., Leviev, G. I. & Ovchinnikova, L. V., Nonlinear microwave
response of YBCO single crystal in constant magnetic field. *J. Exp. Theor. Phys.* **88,** 1229-1235 (1999).

[3] Gallitto, A. A. & Vigni, M. L., Harmonic emission at microwave frequencies in $YBa_2Cu_3O_7$ single crystals near T_c. *Physica C* **305,** 75-84 (1999).

[4] Lee, S.-C. et al. Doping-dependent nonlinear Meissner effect and spontaneous currents in high-T_c superconductors. *Phys. Rev. B* **71**, 014507 (2005)

[5] Mircea, D. I., Xu, H. & Anlage, S. M. Phase-sensitive harmonic measurements of microwave nonlinearities in cuprate thin films. *Phys. Rev. B* **80**, 144505 (2009).

[6] Coffey, M. W., Coupled nonlinear electrodynamics of type-II superconductors in the mixed state. *Phys. Rev. B* **46,** 567(R) (1992).

[7] Coffey, M. W. & Clem, J. R., Unified theory of effects of vortex pinning and flux creep upon the rf surface impedance of type-II superconductors. *Phys. Rev. Lett.* **67,** 386 (1991).

[8] Barišić, N. et al., Demonstrating the model nature of the high temperature superconductor $HgBa_2CuO_{4+\delta}$. *Phys. Rev. B* **78,** 054518 (2008).

[9] Tranquada, J. M. et al., Evidence of unusual superconducting correlations coexisting with stripe order in $La_{1.875}Ba_{0.125}CuO_4$. *Phys. Rev. B* **78,** 174529 (2008).

[10] Chang, J. et al., Decrease of upper critical field with underdoping in cuprate superconductors. *Nat. Phys.* **8,** 751-756 (2012).

[11] Lang, G. et al., Spatial competition of the ground states in 1111 pnictides. *Phys. Rev. B* **94,** 014514 (2016).

[12] Salamon, M. B. & Jaime, M., The physics of manganites: Structure and transport. *Rev. Mod. Phys.* **73,** 583 (2001).

[13] Phillips, J. C., Saxena, A. & Bishop, A. R., Pseudogaps, dopants, and strong disorder in cuprate high-temperature superconductors. *Rep. Prog. Phys.* **66,** 2111-2182 (2003).

[14] Singer, P. W., Hunt, A. W. & Imai, T., ^{63}Cu NQR evidence for spatial variation of hole concentration in $La_{2-x}Sr_xCuO_4$. *Phys. Rev. Lett.* **88,** 047602 (2002).

[15] Bobroff, J. et al., Absence of static phase separation in the high-T_c cuprate $YBa_2Cu_3O_{6+y}$. *Phys. Rev. Lett.* **89,** 157002 (2002).

[16] Rybicki, D. et al., Spatial Inhomogeneities in Single-Crystal $HgBa_2CuO_{4+\delta}$ from ^{63}Cu NMR Spin and Quadrupole Shifts. *J. Supercond. Nov. Magn.* **22**, 179–183 (2009).

[17] Mihajlović, D., Kabanov, V. V. & Müller, K. A., The attainable superconducting T_c in a model of phase coherence by percolating. *Europhys. Lett.* **57,** 254-259 (2002).

[18] Abrikosov, A. A., Possible explanation of the pseudogap in high-temperature cuprates. *Phys. Rev. B* **63,** 134518 (2001).

[19] Carretta, P., Lascialfari, A., Rigamonti, A., Rosso, A. & Varlamov, A. Superconducting fluctuations and anomalous diamagnetism in underdoped $YBa_2Cu_3O_{6+x}$ from magnetization and ^{63}Cu NMR-NQR relaxation measurements. *Phys. Rev. B* **61**, 12420 (2000)

[20] Mosqueira, J., Cabo, L. & Vidal, F., Structural and T_c inhomogeneities inherent to doping in $La_{2-x}Sr_xCuO_4$ superconductors and their effects on the precursor diamagnetism. *Phys. Rev. B* **80,** 214527 (2009).

[21] Phillips, J. C., Percolative theories of strongly disordered ceramic high-temperature superconductors. *Proc. Nat. Acad. Sci. USA* **107,** 1307-1310 (2010).

[22] Ovchinnikov, Y. N., Wolf, S. A. & Kresin, V. Z., Intrinsic inhomogeneities in superconductors and the pseudogap phenomenon. *Phys. Rev. B* **63,** 064524 (2000).

[23] Gomes, K. K. et al., Visualizing pair formation on the atomic scale in the high-T_c superconductor $Bi_2Sr_2CaCu_2O_{8+d}$. *Nature* **447**, 569 (2007).

[24] Honma, T. & Hor, P. H. Quantitative connection between the nanoscale electronic inhomogeneity and the pseudogap of $Bi_2Sr_2CaCu_2O_{8+\delta}$ superconductors. *Physica C* **509,** 11-15 (2015).

[25] Boyer, M. C. et al., Imaging the two gaps of the high-temperature superconductor $Bi_2Sr_2CuO_{6+x}$. *Nat. Phys.* **3,** 802-806 (2007).

[26] Pelc, D., Popčević, P., Yu, Požek, M., G., Greven, M. & Barišić, N., Unusual behaviour of cuprates explained by heterogeneous charge localization. Preprint at http://arxiv.org/abs/1710.10221 (2017).

[27] Barišić, N. et al., Hidden Fermi-liquid behavior throughout the phase diagram of the cuprates. Preprint at http://arxiv.org/abs/1507.07885 (2015).

[28] Grissonnanche, G. et al., Direct measurement of the upper critical field in cuprate superconductors. *Nat. Comm.* **5,** 3280 (2014).

[29] Sonier, J. E. et al., Hole-doping dependence of the magnetic penetration depth and vortex core size in $YBa_2Cu_3O_y$: Evidence for stripe correlations near 1/8 hole doping. *Phys. Rev. B* **76,** 134518 (2007).
